# Prognostic significance of T lymphocyte subgroups (CD4 and CD8) in lung cancer patients after neoadjuvant chemotherapy

**DOI:** 10.1186/s13019-024-02596-z

**Published:** 2024-03-11

**Authors:** Aykut Elicora, Busra Yaprak Bayrak, Cigdem Vural, Huseyin Fatih Sezer, Semra Uzun Erkal, Elif Metin

**Affiliations:** 1 Department of Thoracic Surgery, Kocaeli University Faculty of Medicine, Kocaeli, Turkey; 2 Department of Pathology, Kocaeli University Faculty of Medicine, Kocaeli, Turkey

**Keywords:** CD4 + and CD8 + T lymphocytes, Lung carcinoma, Neoadjuvant chemotherapy, Prognosis, Tumor microenvironment

## Abstract

**Objective:**

The basis for current and future lung cancer immunotherapy depends on our knowledge of molecular mechanisms of interactions between tumor and immune system cells. Interactions that occur between different intratumoral populations of the same cells are important. In our study, we aimed to evaluate relationship between the clinical and prognostic features and T lymphocyte subgroups of patients with lung tumors after neoadjuvant treatment.

**Methods:**

A total of 72 patients were included in our study, including study group, 39 of whom received neoadjuvant chemotherapy. Clinical/radiological/pathological findings of patients and CD4/CD8 staining rates in peritumoral/intratumoral areas were recorded.

**Results:**

Our study revealed significantly lower intratumoral CD4 + T cell density and lower intratumoral CD4/CD8 ratio in primary tumor after neoadjuvant therapy (respectively, 0.012 and 0.016). Considering tumor types, when control-study groups were compared, inflammation was statistically significant only in adenocarcinoma subtype; intratumoral CD4/CD8 ratio was statistically significant only in squamous-cell carcinoma subtype (respectively, *p* = 0.0008 and *p* = 0.0139). When CD4 + T lymphocytes and CD8 + T lymphocytes and CD4/CD8 ratio were compared between control and study groups in low-stage patients according to clinical stages, only intratumoral CD4 + T lymphocyte values and intratumoral CD4/CD8 ratio were significant (respectively, *p* = 0.0291 ve *p* = 0.0154).

**Conclusion:**

All cell types of innate and adaptive intratumoral immunity can affect lung cancer tissues simultaneously, and these interactions have a very complex structure. Understanding the tumor microenvironment and the different roles of associated cancer immune cells may lead to the discovery of new targets for immunological therapies and increased survival times in lung cancer.

## Introduction

Lung cancer is the leading cause of cancer-related deaths globally. Lung cancers have very heterogeneous characteristics at the cellular and histological level. 80–85% of these cases are non-small cell lung cancer (NSCLC) [1]. The heterogeneous structure of the tumor, microenvironmental factors of the tumor, complex molecular properties and limited treatment options affect the prognosis. The main radical treatment for NSCLC is surgery; However, direct surgery is difficult in some patients with NSCLC, especially in stage N2. The use of immune checkpoint inhibitors and tyrosine kinase inhibitors in the treatment of lung cancer in these patients significantly improves the prognosis [2]. Neoadjuvant chemotherapy (at least two cycles of chemotherapy before surgery) is given to these patients to downstage the tumor, improve operability, and eliminate micro-metastatic disease. Neoadjuvant chemotherapy is associated with good prognosis [3] in patients with NSCLC and can reshape the tumor immune microenvironment, which is important in tumor development. According to the literature, in one study, neoadjuvant chemotherapy increased the 5-year overall survival rate from 42 to 58% compared to adjuvant chemotherapy; In another study, it was found to increase from 40 to 45%. And it has been suggested that it significantly improves overall survival rates [4–6].

The basis for current and future lung cancer immunotherapy depends on our knowledge of the molecular mechanisms of interactions between tumor and immune system cells, as well as the interactions that occur between different intratumoral populations of the same cells. CD4 + T and CD8 + T cells constitute major components of tumor-infiltrating lymphocytes (TIL) and play different roles in antitumor immunity; they also consist of different cell subtypes. CD4 + T cells have high functional heterogeneity and have been reported to have different prognostic values in NSCLC. However, overall, high CD8 + T cell infiltration is a favorable prognostic factor for NSCLC [6–8]. According to several studies, different tumor-infiltrating lymphocyte phenotypes have been discovered to correlate with the tumor’s response to immune checkpoint inhibitors [9, 10]. Patients with NSCLC receiving neoadjuvant chemotherapy followed by surgery were found to have higher infiltrating levels of epithelial CD3 + CD4 + T lymphocytes and CD68 + epithelial and stromal tumor-associated macrophages than patients with prior surgery [5]. It has been reported that neoadjuvant chemotherapy can reshape the tumor immune microenvironment and increase cytotoxic T cells and tissue-resident memory T cell infiltration [5–11].

In our study, we examined the infiltrating level of TIL, CD4 + TIL and CD8 + TIL in patients with lung tumors operated without neoadjuvant therapyand patients resected after neoadjuvant treatment, and we aimed to evaluate the relationship between their clinical and prognostic features and lymphocyte subgroups.

## Materials and methods

### Study group and data collection

In our study, 33 patients who received neoadjuvant therapyfor lung carcinoma and underwent resection and were diagnosed with lung malignancy between January 2008 and November 2022, and 39 patients who were diagnosed with lung carcinoma and operated without receiving neoadjuvant therapywere included in the study as a control group. Patients who were under 18 years of age, did not have sufficient examination and clinical data, were not diagnosed with NSCLC, had an accompanying immune system disease, had an infection or was using a drug that affects the immune system were excluded from the study. The cases were evaluated in terms of age, gender, smoking, positron emission tomography, tumor localization, tumor size, operation type, preoperative diagnosis and diagnostic method, postoperative diagnosis, chemotherapy, radiotherapy, surveillance, recurrence, metastasis and tumor stage. All cases receiving neoadjuvant chemotherapy were treated with cisplatin and gemcitabine.

### Histopathological analysis

**Fig. 1:**
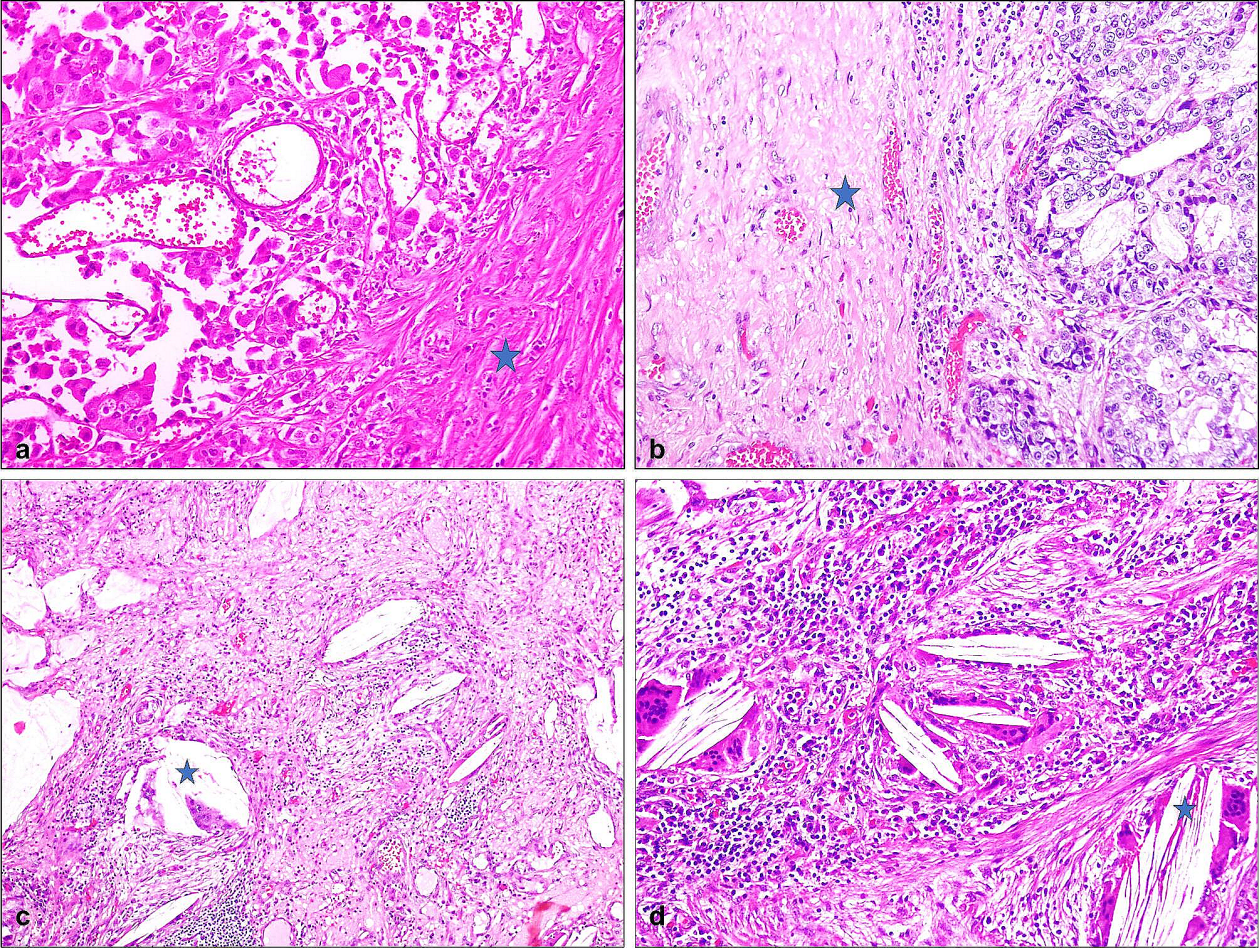
**a + b.** Fibrosis densities in a case diagnosed with adenocarcinoma receiving neoadjuvant treatment (H&E, x400), **c + d.** Granulation tissue containing dense foreign body-style giant cells (marked with an asterisk) in a case diagnosed with adenocarcinoma receiving neoadjuvant therapy(H&E, x100 ve x200)

NSCLC cases were retrospectively reviewed in the pathology laboratory. For each patient, tumor type, tumor dimensions, residual tumor rate if neoadjuvant chemotherapy was received, lymphovascular invasion, pleural invasion, bronchial surgical margin invasions, inflammation, necrosis, fibrosis, foreign body reactions, and lymph node metastases were recorded (Fig. 1). The 8th staging system of the International Association for the Study of Lung Cancer (IASLC) was used for tumor staging of all patients [12]. Tumor-infiltrating lymphocyte in the intratumoral and peritumoral region were evaluated by 2 senior pathologists under an Olympus BX50 microscope. The degree of TIL infiltration was evaluated at the time of diagnosis using a minimum of three hematoxylin-eosin sections. The blocks with the most intense tumor and inflammation were selected for immunohistochemical analysis.

### Immunohistochemical analysis

**Fig. 2:**
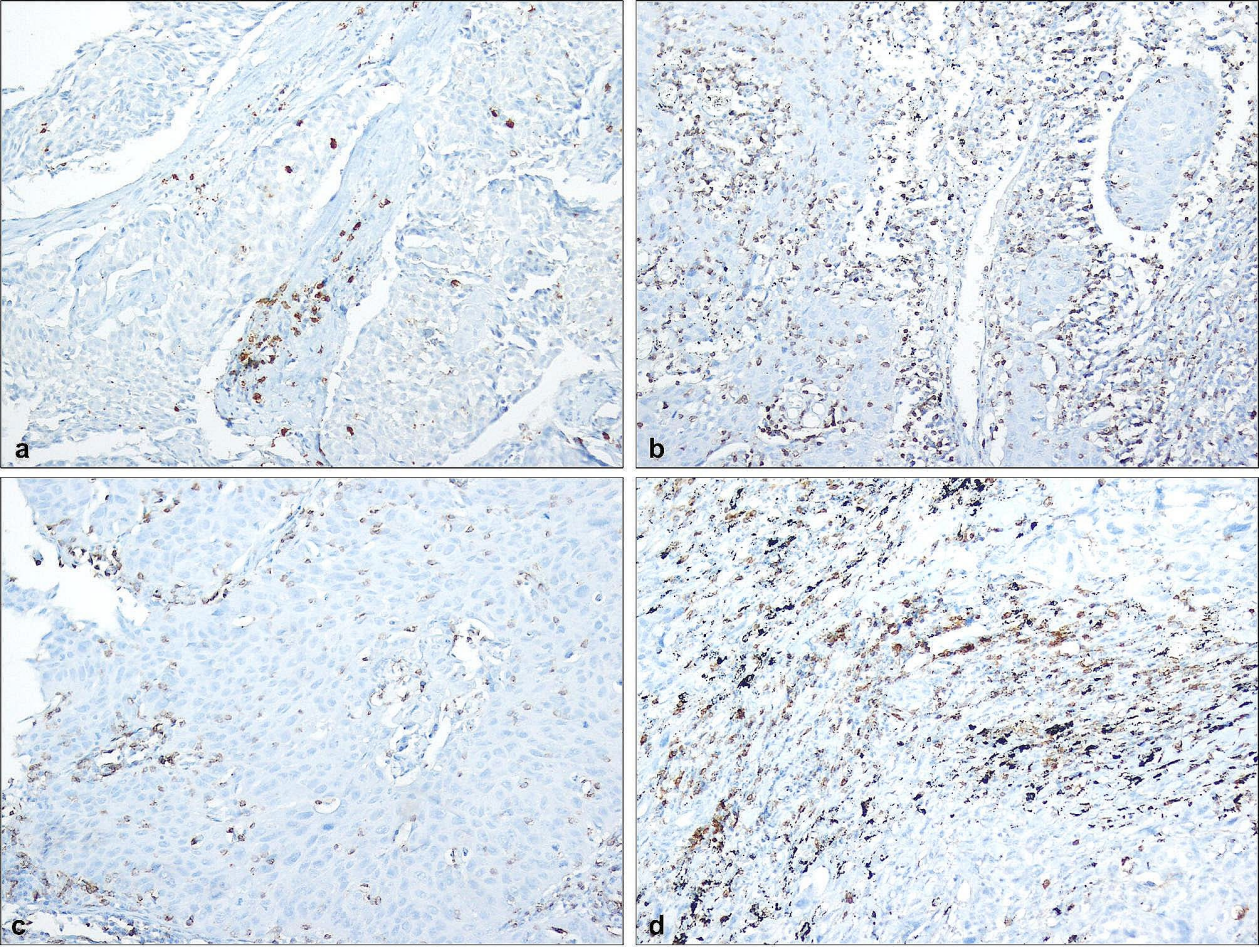
**a + b.** CD4 staining intensities in a case diagnosed with squamous cell carcinoma receiving neoadjuvant therapy(CD4, x200), **c + d.** CD8 staining intensities in cases diagnosed with squamous cell carcinoma receiving neoadjuvant therapy(CD8, x200)

Immunohistochemical staining was performed using CD4 (Ventana, SP35) and CD8 (Ventana, SP57) antibodies on 4 μm thick sections from paraffin blocks containing lung resection materials (Fig. 2). Staining was done on a Ventana automatic device (RocheVentana), using Ventana Optiview and Ultraview DAB kits. Tonsil tissue was used for all two markers as a positive external control.

### Scoring system

In patients receiving both control and neoadjuvant chemotherapy, the TIL infiltration levels were classified as low, middle, or high using the IASLC Neoadjuvant Pathology Recommendations [12]. The main pathologic response was defined as the number of residual tumor cells in the tumor bed ranging from 0 to 10%. The residual tumor cells were evaluated and recorded from 0 to 100% at 10% intervals. The staining of CD4 and CD8 in lymphocytes in and around the tumor was evaluated in the most intense area. CD4 or CD8 positive lymphocytes in one high magnification field were recorded as a percentage. Then, the CD4/CD8 ratio was found in both intratumoral and peritumoral areas. These values were measured both in patients who underwent surgery without chemotherapy and in patients who underwent surgery after neoadjuvant chemotherapy. In addition, these values were compared with low and high grades in patients receiving neoadjuvant chemotherapy.

### Statistical analysis

All statistical analyzes were performed using IBM SPSS for Windows version 20.0 (IBM Corp., Armonk, NY, USA). The Shapiro-Wilk test was used to evaluate the assumption of normality. Continuous variables are presented as mean ± standard deviation or (in case of non-normal distribution) median (interquartile range) based on normal distribution. Comparisons between groups were made using the Mann-Whitney U test. Relationships between categorical variables were determined using the Chi-square test. Kaplan-Meir method with Log-Rank test was used for survival analysis. p-value < 0.05 was considered statistically significant.

## Results

The majority of our study was male (89.9%) and the average age was 61 (20–84). Smoking history was around 87.5%. Tumor locations were mostly in the upper lobe of the right lung in both groups (40.3%). If there was a tumor in the left lung, the probability of finding it in the upper lobe was higher (31.9%). Tru-cut or bronchoscopic biopsy was mostly used as a preoperative diagnostic method (59.7%). When positron emission tomography was evaluated, values were mostly found to be between 10 and 15 in terms of standardized uptake value (34.7%). However, no statistical significance was detected between the two groups for these data (*p* > 0.05) (Table 1). The type of surgery mostly performed on patients was lobectomy, but there was no difference between the two groups (*p* = 0.084) (Table 1).

**Table 1:** Demographic, clinical and histopathological data of the patients, and comparison of these data according to the control and study groups

	Total (*N* = 72)	Control Group (*n* = 39)	Study Group (*n* = 33)	*P* value
Gender, *N* (%)				0.456
Male	64 (89.9)	36 (92.3)	28 (84.8)	
Female	8 (11.1)	3 (7.7)	5 (15.2)	
Age, $\bar x$ (Min-Max)	61 (20–84)	63 (41–84)	59 (20–77)	0.078
Smoking, *N* (%)	63 (87.5)	35 (89.7)	28 (84.8)	0.723
Location, *N* (%)				0.175
Right Lung,				
superior lob	29 (40.3)	15 (38.5)	14 (42.4)	
median lob	3 (4.2)	2 (5.1)	1 (3.0)	
inferior lob	8 (11.1)	6 (15.4)	2 (6.1)	
Left Lung,				
superior lob	23 (31.9)	12 (30.8)	11 (33.3)	
inferior lob	9 (12.5)	4 (10.2)	5 (15.2)	
Preoperative diagnostic method, *N* (%)				0.719
Aspiration	29 (40.3)	14 (35.9)	15 (45.5)	
Biopsy	43 (59.7)	25 (64.1)	18 (54.5)	
SUV value with CT-PET				0.530
<5	6 (8.3)	5 (12.8)	1 (3.0)	
5–10	15 (20.8)	7 (17.9)	8 (24.2)	
10–15	25 (34.7)	14 (35.9)	11 (33.3)	
15–20 >20	18 (25.0) 8 (11.1)	10 (25.6) 3 (7.7)	8 (24.2) 5 (15.2)	
Surgery type, *N* (%)				0.084
Lobectomy	44 (61.1)	29 (74.4)	15 (45.5)	
Bilobectomy	5 (6.9)	2 (5.1)	3 (9.1)	
Segmentectomy	4 (5.6)	1 (2.6)	3 (9.1)	
Pneumonectomy	19 (26.4)	7 (17.9)	12 (36.4)	
Tumor subtype, *N* (%)				0.064
Adenocarcinoma	33 (45.8)	19 (48.7)	14 (42.2)	
SCC	29 (40.3)	18 (46.2)	11 (33.3)	
NSCLC	10 (13.9)	2 (5.1)	8 (24.2)	
TNM Stage, *N* (%)				**0.004**
Stage 1	25 (34.7)	16 (41.0)	9 (27.3)	
Stage 2	23 (31.9)	13 (33.3)	10 (30.3)	
Stage 3	10 (13.9)	8 (20.5)	2 (6.1)	
Stage 4	14 (19.4)	2 (5.1)	12 (36.4)	
Tumor Diameter (mm) $\bar x$ (Min-Max)	40.0 (7.0–80.0)	35 (13.0–80.0)	40 (7.0–73.0)	0.317
Lymph node metastasis, *N* (%)	29 (40.3)	15 (38.4)	14 (42.4)	0.432
Pleural invasion, *N* (%)	11 (15.3)	6 (15.4)	5 (45.5)	1.000
Lymphovascular invasion, *N* (%)	14 (19.4)	9 (23.1)	5 (15.2)	0.584
Fibrosis, *N* (%)	51 (70.8)	23 (59.0)	28 (84.8)	**0.032**
Necrosis, *N* (%)	42 (58.3)	25 (64.1)	17 (51.5)	0.401
Foreign body reaction, *N* (%)	11 (15.3)	0 (0.0)	11 (33.3)	**0.000**
Degree of inflammation, *N* (%)				**0.000**
Mild	27 (37.5)	16 (41.0)	11 (33.3)	
Moderate Severe	27 (37.5) 18 (25.0)	20 (51.3) 3 (7.7)	7 (21.2) 15 (45.5)	
CD4 + T lymphocyte, $\bar x$ (Min-Max)				**0.012** 0.233
Intratumoral	17 (0-220)	22 (0–87)	11 (2-220)	
Peritumoral	158.5 (1-405)	157 (1-357)	214 (22–405)	
*P* value	**< 0.0001**	**< 0.0001**	**< 0.0001**	
CD8 + T lymphocyte, $\bar x$ (Min-Max)				
Intratumoral	18 (2-180)	21 (4-180)	18 (2-131)	0.635
Peritumoral	95 (12–374)	157 (1-357)	127 (12–374)	0.156
*P* value	**< 0.0001**	**< 0.0001**	**< 0.0001**	
CD4/CD8 ratio, $\bar x$ (Min-Max)				
Intratumoral	0.87 (0-7.12)	1.33 (0.0-7.13)	0.77 (0.11–2.67)	**0.016**
Peritumoral	1.49 (0.03–11.42)	1.50 (0.03–11.42)	1.46 (0.18–6.50)	0.079
*P* value	**0.001**	0.113	**0.0014**	
Radiotherapy, *N* (%)	24 (33.3)	12 (30.8)	12 (36.4)	0.802
Survival, X ± SD (month)	52.3 ± 4.1	48.9 ± 3.4	57.1 ± 5.2	0.501
Relapse, N (%)	12 (16.7)	6 (15.4)	6 (18.2)	1.000
Distant Organ Metastasis, *N* (%)	10 (13.9)	4 (10.3)	6 (18.2)	0.496

$\bar x$*(Min-Max): Median (Minimum-Maximum), X ± SD: Mean ± Standard deviation, CT-PET: Computed Tomography-Positron Emission Tomography, SUV*: standardized *uptake value*

When evaluated in terms of survival, the estimated life expectancy in the control group was found to be 48.9 ± 3.4 months; the estimated life expectancy in the group receiving neoadjuvant therapywas found to be 57.1 ± 5.2 months. There was no statistically significant difference in life expectancy between both groups (*p* = 0.928). At the same time, no statistically significant difference was found when radiotherapy reception was evaluated in terms of recurrence and distant organ metastasis (respectively, *p* = 0.802, *p* = 1.000, *p* = 0.496) (Table 1).

In preoperative examinations, the tumor subtype was adenocarcinoma with a rate of 45.8% and squamous-cell carcinoma (SCC) with a rate of 40.3%. No tumor was found in 5 of these patients after neoadjuvant treatment. Ten patients were diagnosed with NSCLC, not otherwise specified (NOS). Two patients were in the control group and both of them were diagnosed with SCC after the operation. The other 8 patients were in the neoadjuvant therapygroup and 4 patients were diagnosed with adenocarcinoma, 1 patient with SCC, and 3 patients with large cell carcinoma. However, there was no significant difference between groups (*p* = 0.064) (Table 1). When we look at the TNM staging of all patients, most of them were stage 1 and 2 (34.7% and 31.9%). The difference between groups was statistically significant (*p* = 0.004) (Table 1).

When evaluated morphologically in terms of pleural invasion, lymphovascular invasion, bronchial surgical margin invasion and necrosis, no statistically significant difference was found between the groups (respectively, *p* = 1.000, *p* = 0.584, *p* = 0.401). However, as expected, fibrosis, foreign body reaction and inflammation were observed to be higher in the group receiving neoadjuvant therapy and these differences were found to be significant (respectively, *p* = 0.032, *p* = 0.000, *p* = 0.000) (Table 1). And also there was no significant difference in tumor relapse times between the control and study groups (*p* < 0.05). Relapse times were between the 23rd and 41st months after surgery.

The number of CD4 + T lymphocytes in the peritumoral area was slightly higher in the study group compared to the control group, but the difference was not significant (*p* = 0.233). This number in the intratumoral area was observed to be lower in the study group, and this rate was found to be statistically significant when evaluated between groups (*p* = 0.012). The numbers of CD8 + T lymphocytes in both peritumoral and intratumoral areas were slightly lower in the study group compared to the control group, but the difference was insignificant (respectively, *p* = 0.635, *p* = 0.156). Peritumoral CD4/CD8 ratio was also slightly lower in the study group, but this difference was also not significant (*p* = 0.079). Intratumoral CD4/CD8 ratio was lower in the study group compared to the control group and this difference was found to be statistically significant (*p* = 0.016) (Table 1).

Intratumoral CD4 + T, CD8 + T lymphocyte values, CD4/CD8 ratio and peritumoral CD4+, CD8 + T lymphocyte values, CD4/CD8 ratio, when groups were evaluated within themselves, peritumoral values and ratios were found to be higher than intratumoral values and ratios and the difference between them was statistically significant (*p* < 0.05). No significance was found between intratumoral and peritumoral CD4/CD8 ratios only in the control group (*p* = 0.113) (Table 1).

When we compared the histopathological data of the cases according to tumor subtype, no statistical difference was detected in terms of tumor size, lymph node metastasis, pleural invasion and lymphovascular invasion, necrosis between the control and study groups within each subtype (*p* > 0.05). However, fibrosis was observed in the SCC subtype study group; Foreign body reaction was higher in the adenocarcinoma subtype study group and this difference was significant (respectively, *p* = 0.0038 and *p* = 0.007). It was noted that there was a statistically significant difference for inflammation between the control and study group with the adenocarcinoma subtype and for intratumoral CD4/CD8 ratio between the control and study group with SCC subtype (respectively, *p* = 0.0008 ve *p* = 0.0139) (Table 2).

**Table 2:** Comparison of histopathological data of cases according to tumor subtype (*n* = 72)

	Adenocarcinoma (*n* = 33)			SCC (*n* = 29)			NSCLC (*n* = 10)		
	Control group (*n* = 19)	Study group (*n* = 14)	*P* value	Control group (*n* = 18)	Study group (*n* = 11)	*P* value	Control group (*n* = 2)	Study group (*n* = 8)	*P* value
Tumor Diameter (mm) $\bar x$ (Min-Max)	35 (15–80)	40 (12–73)	0.956	35.5 (13–65)	40 (7–55)	0.421	30 (15–45)	36.5 (5–70)	0.711
Lymph node metastasis, *N* (%)	10 (52.6)	8 (57.1)	0.797	5 (27.8)	4 (36.4)	0.943	0 (0.0)	2 (25.0)	0.429
Pleural invasion, *N* (%)	3 (15.8)	2 (14.3)	0.905	2 (11.1)	0 (0.0)	0.696	1 (50.0)	3 (37.5)	0.747
Lymphovascular invasion, *N* (%)	6 (31.6)	3 (21.4)	0.801	3 (16.7)	0 (0.0)	0.423	0 (0.0)	2 (25.0)	0.429
Fibrosis, *N* (%)	15 (78.9)	12 (85.7)	0.967	7 (38.9)	11 (100.0)	**0.0038**	1 (50.0)	5 (62.5)	0.747
Necrosis, *N* (%)	11 (57.9)	6 (42.9)	0.616	13 (72.2)	6 (54.5)	0.569	1 (50.0)	2 (25.0)	0.490
Foreign body reaction, *N* (%)	0 (0.0)	6 (42.9)	**0.007**	0 (0.0)	3 (27.3)	0.087	0 (0.0)	2 (25.0)	0.429
Degree of inflammation,									
*N* (%)									
Mild	6 (31.6)	3 (21.4)		9 (50.0)	6 (54.5)		1 (50.0)	2 (25.0)	
Moderate	11 (57.9)	1 (7.1)	**0.0008**	8 (44.4)	3 (27.3)	0.447	1 (50.0)	3 (37.5)	0.564
Severe	2 (10.5)	10 (71.4)		1 (5.6)	2 (18.2)		0 (0.0)	3 (37.5)	
CD4/CD8 ratio									
$$\bar x$$ (Min-Max)									
Intratumoral	1.13 (0.00-7.13)	0.74 (0.13–2.67)	0.469	1.52 (0.04–5.60)	0.77 (0.11-2.00)	**0.0139**	1.2 (0.33–2.07)	0.67 (0.22–2.05)	0.794
Peritumoral	1.47 (0.03–11.42)	1.37 (1.02–6.50)	0.702	1.66 (0.23–6.76)	1.17 (0.18–3.85)	0.173	0.59 (0.42–0.75)	1.60 (0.39–3.35)	0.267
*P* value	0.093	0.094		0.548	0.114		> 0.999	**0.0499**	

*SCC: Squamous cell carcinoma, NSCLC: Non-small cell-lung carcinoma,*$\bar x$* (Min-Max): Median (Minimum-Maximum)*,

When intratumoral CD4/CD8 ratio and peritumoral CD4/CD8 ratio were evaluated within the groups, statistical significance was observed only in study group with NSCLC subtype (*p* = 0.0499) (Table 2).

When the CD4 + T lymphocyte and CD8 + T lymphocyte values and CD4/CD8 ratio were compared between the control and study groups in low-stage patients according to clinical stages, it was noted that only the difference between intratumoral CD4 + T lymphocyte values and intratumoral CD4/CD8 T lymphocyte ratios was significant (respectively, *p* = 0.0291 ve *p* = 0.0154) (Table 3).

**Table 3:** Comparison of CD4^+^ T lymphocyte and CD8^+^ T lymphocyte rates of the clinical stages (*n* = 72)

	Low Stage Median (Minimum-Maximum)			High Stage Median (Minimum-Maximum)		
	Control group (*n* = 29)	Study group (*n* = 19)	*P* value	Control group (*n* = 10)	Study group (*n* = 14)	*P* value
CD4 + T lymphocyte						
Intratumoral	22 (0–87)	14 (3–48)	**0.0291**	26.5 (1–78)	8.5 (2-220)	0.266
Peritumoral	157 (40–357)	186 (27–378)	0.238	179.5 (1-352)	264.5 (22–405)	0.458
*P* value	**< 0.0001**	**< 0.0001**		**0.0021**	**< 0.0001**	
CD8 + T lymphocyte						
Intratumoral	21 (4-180)	18 (3–57)	0.899	16.5 (9–42)	15.5 (2-131)	0.429
Peritumoral	82 (18–360)	98 (22–330)	0.493	76.5 (20–245)	192 (12–374)	0.079
*P* value	**< 0.0001**	**0.0002**		**0.0092**	**0.0003**	
CD4^+^/CD8^+^T ratio						
Intratumoral	1.33 (0-7.13)	0.57 (0.14–2.70)	**0.0154**	1.47 (0.90–4.33)	1.40 (0.11-	0.564
Peritumoral	82 (18–360)	1.51 (0.18–3.85)	0.598	1.58 (0.03–7.04)	2.50) 1.24 (0.38–6.50)	0.472
*P* value	0.200	**0.0001**		0.276	> 0.999	

When intratumoral CD4 and CD8 values and peritumoral CD4 and CD8 values were evaluated within the groups in both low and high stage patients, it was determined that peritumoral values and ratios were higher than intratumoral values and ratios in both the control and study groups and the difference between them was statistically significant (*p* < 0.05). When the groups were evaluated within themselves in low-stage patients according to clinical stages, it was observed that the difference between intratumoral CD4/CD8 ratio and peritumoral CD4/CD8 ratio was significant only in the study group (*p* = 0.0001) (Table 3).

**Fig. 3:**
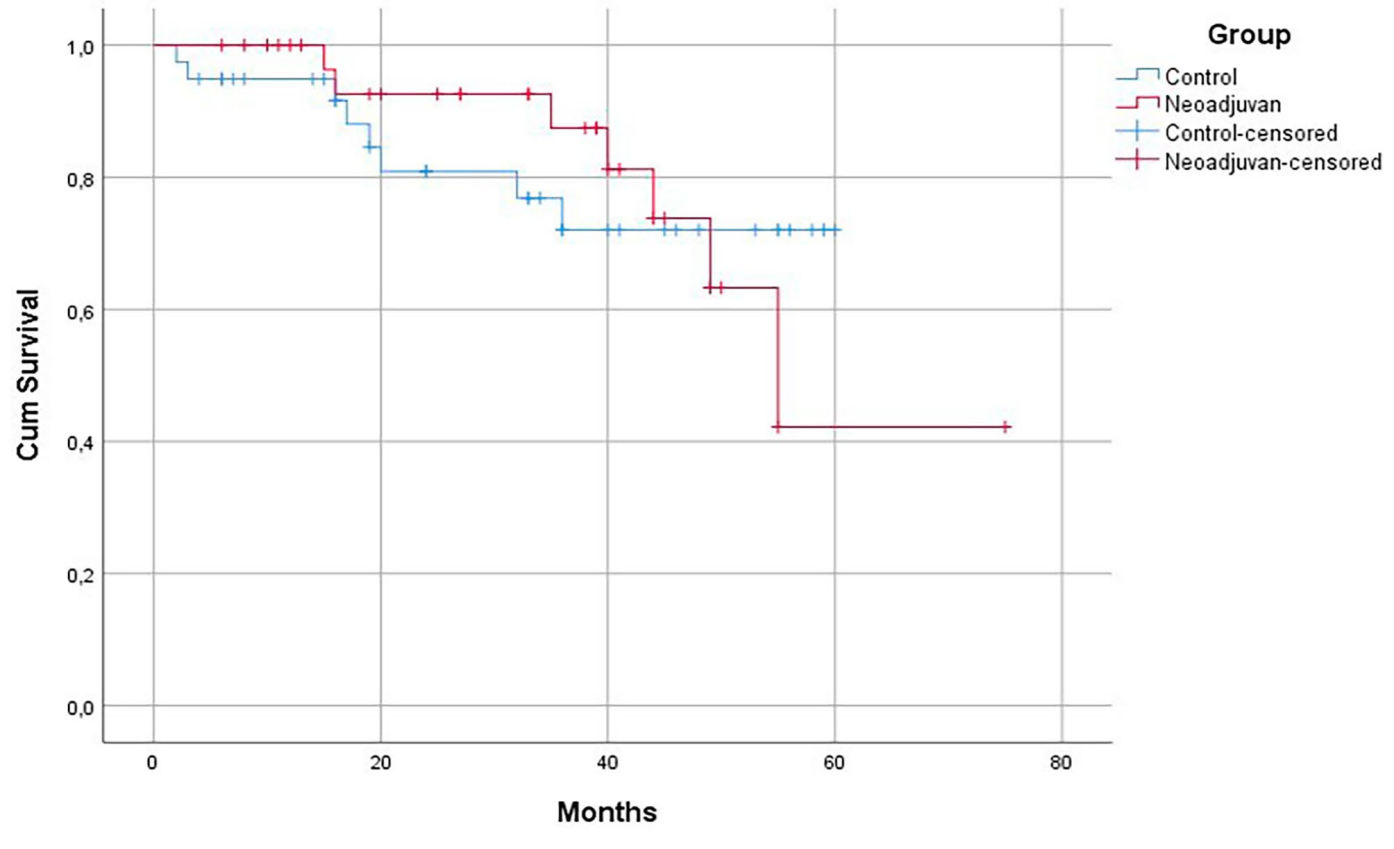
Kaplan–Meier survival analysis of the control group and study group recievining nedoadjuvant chemotherapy

Kaplan–Meier survival analysis found that the patients receiving neoadjuvant chemotherapy did not show a significant survival benefit compared to the control group (*p* = 0.928, 95% CI: 52.47–65.92) (Fig. 3).

## Discussion

This retrospective study compared the density of CD4 + and CD8 + T cells in the peritumoral and intratumoral area of patients with NSCLC operated with and without neoadjuvant treatment, and their relationships with each other according to tumor subtype and tumor stage. The results showed that the morphological inflammation intensities were severe in 45.5% of the patients received neoadjuvant therapywhen compared to patients not received therapy. We revealed significantly lower intratumoral CD4 + T cell density and lower intratumoral CD4+/CD8 + ratio in the primary tumor of the patients received neoadjuvant therapy. Peritumoral CD4 + T cell density and, intratumoral and peritumoral CD8 + T cell densities and peritumoral CD4+/CD8 + ratio were slightly lower in patients received neoadjuvant treatment, but these were not statistically significant. Comparing the subgroups of tumor, the inflammation was significantly higher only in the patients with adenocarcinoma subtype receiving neoadjuvant therapy. The intratumoral CD4+/CD8 + ratio was significantly different in the SCC tumor subtype. Comparing the CD4 + T and CD8 + T lymphocytes according to clinical stages, it was noted that the intratumoral CD4 + T lymphocyte rate and CD4+/CD8 + T lymphocyte ratios significantly lower in patients with low stage NSCLC receiving neoajuvant therapy.

Tumor tissue is not only a mass consisting of malignant cancer cells, but also consists of stromal cells, fibroblasts, endothelial cells, blood vessels, lymphatic vessels, immune cells and various signaling molecules (cytokines, chemokines) surrounding the tumor cells [13]. In this structure, defined as the tumor microenvironment, each cell has its own unique structure and shows different metabolic properties. It has been reported that the tumor microenvironment has an impact on tumor development, prognosis, treatment modalities and development of resistance to treatment [14]. It is thought that immune cells play an important role in these interactions, and innate immune cells and adaptive immune cells (T cells and B cells) contribute to the development of tumor and metastasis when they are in the tumor microenvironment [15, 16]. Chemotherapy applied to cancers stimulates apoptosis and leads to immune system-mediated cell death [16]. Neoadjuvant chemotherapies suppress immunity; However, in current studies, it is accepted that some chemotherapy agents can regulate and modulate antitumor immunity. It has been observed that cell deaths, especially those occurring after chemotherapy, have the potential to trigger tumor-related immunity [16, 17]. In our study, we comprehensively analyzed the relationships of T lymphocyte subgroups in the tumor microenvironment with clinical features in lung cancer patients receiving neoadjuvant chemotherapy. We observed that the patients who received neoadjuvant chemotherapy had higher percentages of peripheral CD4 + T cells but lower percentages of peripheral CD8 + T cells than those who did not receive neoadjuvant chemotherapy. These findings suggest a potential prognostic significance of T lymphocyte subgroups in the context of neoadjuvant chemotherapy for lung cancer. To further assess the prognostic significance of neoadjuvant therapy, we conducted survival analyses to examine the correlation between the survival outcomes of groups. Survival analyses revealed that there is no significant difference in the cumulative survival duration of the patient receiving a neoadjuvant therapy and not received this therapy.

Tumor cells are in close relationship with the microenvironment that surrounds them. They can make changes in the local metabolic environment through various means and ensure their survival and development through extracellular signals. Some of the traditional chemotherapeutic agents rearrange the cellular location, making dying tumor cells visible to the immune system. Other agents act by initiating a temporary lymphocyte destruction, disrupting immunosuppressive mechanisms, or by direct or indirect stimulatory signals on immune effectors [16]. Changes in the components of this tumor microenvironment affect the immunity developed against the tumor [17]. Previous studies suggest that neoadjuvant chemotherapy plays an important role in antitumor immunity by making changes in the tumor microenvironment [3]. In addition to the decrease in viable tumor cells and tumor immune cell infiltration, proliferative fibrosis, which is thought to reflect immune activation in the host, is also observed in resection specimens after neoadjuvant chemotherapy [18]. The presence of mature fibrosis is associated with the ongoing cytotoxic effect after neoadjuvant chemotherapy and is seen as an indicator of good prognosis [12, 19]. In our study, no tumor was found in five of the patients who received neoadjuvant therapyin the examination performed after surgical resection. Comparing groups receiving and not receiving neoadjuvant treatment, we observed that severe inflammation and fibrosis were more common in the neoadjuvant therapygroup, with statistically significant differences. It is important to note that the control group included patients with stage 4 lung cancer who underwent surgery without receiving neoadjuvant treatment. The decision for surgery without neoadjuvant therapy in this subgroup was based on the clinical considerations, patient-specific factors, or challenges in the radiological examination. While this introduces a potential source of heterogeneity, it reflects the real-world clinical scenario where surgery without neoadjuvant may be considered for certain patients with advanced disease. The inclusion of this subgroup allows for a more comprehensive understanding of the effects of neoadjuvant therapy on the tumor microenvironment in the context of different disease stages.

Although the clinical response to neoadjuvant chemotherapy varies greatly depending on the tumor type and the treatment regimen applied, it is still not at the desired level due to the clinical and biological heterogeneity of lung tumors [20]. Survival rates after neoadjuvant chemotherapy regimens with platinum-based agents were found to be only 5.4% higher than in cases undergoing surgery alone. It has been observed that even after complete resection, approximately 60% of stage IIIA patients may develop recurrence after 3 years. Recently, combinations of neoadjuvant chemotherapy and immunotherapy have come to the fore as a powerful treatment alternative due to both the undesirable side effects of neoadjuvant chemotherapy and the developing resistance to treatment and associated relapses. In addition to the cumulative antitumor effect, it is desired to benefit from the synergistic effect of the two treatment modalities in clinical practice, as it makes the tumor microenvironment more suitable for immunotherapy [21]. When we look at the tumor types in our study, when the control-study group is compared, inflammation is only in the adenocarcinoma subtype; intratumoral CD4/CD8 ratio was observed to be statistically significant only in the SCC tumor subtype. In the fibrosis SCC subtype study group; Foreign body reaction was higher in the adenocarcinoma subtype study group and this difference was significant. According to clinical stages, the difference between intratumoral CD4 + T lymphocyte values and intratumoral CD4/CD8 ratios between the control and study groups was statistically significant in low-stage patients.

In evaluating the prognostic importance of immune cells, the cell type, functional status and distribution in the tumor tissue, rather than the absolute number of cells, are the determining factors and have been shown to affect clinical outcomes in cancer patients [17, 22]. In non-small cell lung cancer, approximately two-thirds of the inflammatory cells are lymphocytes, and approximately 80% of the lymphocytes are T cells [23]. Lymphocytes from immune cells can be localized in the tumor center and tumor stroma (intratumoral lymphocytes) as well as at the invasive border of the tumor (peritumoral lymphocytes) [23, 24]. The fact that immune cell infiltration varies according to the stage of cancer suggests that the tumor microenvironment has an important contribution to carcinogenesis and prognosis of the disease. This has accelerated studies on the development of anticancer treatments targeting the tumor microenvironment [25]. These studies conducted to date have shown that high density of CD8 + T lymphocytes, which have cytotoxic functions in many types of cancer, including lung cancer, is associated with tumor cell apoptosis and that it helps suppress cancer progression. It shows that it plays an important role and has a positive effect on prognosis on overall survival and progression-free survival [12, 23, 26]. Some studies have shown that adequate immune infiltration and CD8 + T cell differentiation are also important in suppressing the development of metastasis [27]. Additionally, it has been reported that tumor tissue-resident memory CD8 + T cells are protective against cancer development by secreting various cytokines and/or triggering tumor cell death to maintain tumor-immune balance [11]. Although there are various subtypes of CD4 + T cells, these cells have different and even opposing effects that suppress or stimulate tumor development [1, 7, 11, 23]. According to the study by Chen et al., TILs were found to increase after neoadjuvant therapy. This was reported to be potentially associated with improved clinical outcomes over neoadjuvant immunotherapy or neoadjuvant chemotherapy alone [7]. In this study, we examined the peritumoral and intratumoral T lymphocyte values and ratios in patients with NSCLC receiving neoadjuvant therapy. We found that CD4 + T lymphocyte numbers were significantly higher only and the intratumoral CD4+/CD8 + ratio was significantly lower in the patients receiving neoadjuvant chemotherapy. The reason for this difference may be that the tumor subtype of the majority of our patients was adenocarcinoma. The majority of our patients were low stage. And as the control group, we chose not the tru-cut biopsies of the same patients, but the patients who underwent surgical resection without neoadjuvant chemotherapy to evaluate the T lymphocyte rates in larger tissues.

Studies in the literature have reported that neoadjuvant chemotherapy causes an increase in cytotoxic CD8 + T cell infiltration compared to cases that only underwent surgery [15, 18]. Parra et al. found that there was an increase in the infiltration of epithelial CD4 + T lymphocytes in patients who underwent surgery after neoadjuvant chemotherapy [17]. Wakabayashi et al. reported that stromal CD4 + cells are an indicator of good prognosis [28]. Hiraoka et al. showed that CD8 + and CD4 + T lymphocytes were associated with better survival only when simultaneously present in the tumor epithelium [29]. In another study, it was shown that low CD4 + T lymphocyte, low CD4+ / CD8 + T lymphocyte ratio before neoadjuvant chemotherapy and high CD4 + T lymphocyte, high CD8 + T lymphocyte count after chemotherapy were associated with good prognosis [18]. And although studies conducted in connection with T lymphocyte types have shown that complete resections after neoadjuvant chemotherapy in lung cancer have a positive effect on survival, it has been stated that the rate of complete resection performed does not show a full correlation with the survival rate. This survival rate was thought to be negatively affected by the presence of undetected micrometastases [30]. In our study, when lung cancer cases that received neoadjuvant chemotherapy were compared with lung cancer cases that were primarily operated on, it was found that the peritumoral CD4 + T cell and CD8 + T cell density was higher in the cases that received neoadjuvant chemotherapy, and the intratumoral CD4 + T cell density was higher in the cases that only underwent surgery. Additionally, no tumor was detected in the surgical resection material in patients received neoadjuvant chemotherapy, but 12 patients were at stage 4 according to the TNM stage. When the two groups were compared in terms of survival, although the estimated survival was numerically higher in the cases receiving neoadjuvant treatment, no statistically significant difference was observed.

In conclusion, our study provides valuable insights into the relationship between T lymphocyte subgroups and clinical outcomes in lung cancer patients undergoing neoadjuvant chemotherapy. The survival analyses conducted in this study indicate a potential prognostic significance of CD4 + and CD8 + T cells in the context of neoadjuvant treatment. Further investigations and clinical trials involving neoadjuvant chemotherapy-immunotherapy combinations are warranted to validate and extend these findings, with the ultimate goal of improving treatment approaches and patient outcomes in lung cancer.

## Data Availability

All data generated or analyzed during this study are included in this published article.

## Abbreviations

IASLC: International Association for the Study of Lung Cancer
NSCLC: Non-small cell lung cancer
TIL: Tumor-infiltrating lymphocytes
SCC: Squamous-cell carcinoma
